# Supplementary material for: Are carbon emissions trading and green financial instruments synergistic? -Comprehensive quantitative research based on content analysis
Source: PLoS One. 2024 Mar 7;19(3):e0298601. doi: 10.1371/journal.pone.0298601 (PMC10919601; doi:10.1371/journal.pone.0298601)
Supplement: S1 Data — (ZIP) [file pone.0298601.s001.zip › Data/地级市Z_F工作报告.zip!/╣π╬≈/│τ╫≤╩╨/└┤╫╘╣1⁄2╓┌║┼í╛┬φ┐╦╩2╛▌═°í┐_2016.docx]

各位代表：

现在，我代表市人民政府，向大会作政府工作报告，请予审议，并请市政协委员和其他列席会议的同志提出意见。

一、 2015年和“十二五”工作回顾

2015年，面对经济下行压力持续加大的不利影响，我们坚决贯彻落实中央、自治区和市委关于稳增长、促改革、调结构、惠民生、防风险各项决策部署，主动适应新常态，努力化解下行压力，保持了经济社会持续健康发展，经济运行总体平稳、稳中有进、稳中向好。初步统计，全市地区生产总值增长8%，财政收入增长2.7%，固定资产投资增长26.1%，规模以上工业增加值增长7.7%，外贸进出口总额增长16.9%，社会消费品零售总额增长10.1%，城镇居民可支配收入增长6.4%，农民人均纯收入增长7.8%。一年来，我们狠抓重点、突破难点，推动经济社会发展呈现新特点、新亮点。一是外贸逆势增长，进出口总额占全区近四成，保持全区第一，实现了外贸进出口总额、边境贸易成交额两个1000亿元人民币的目标；二是投资拉动持续有力，固定资产投资增速高于全区8.3个百分点，位居全区第一；三是左江花山岩画文化景观申报世界文化遗产作为2016年中国唯一申报项目，顺利通过国际专家组现场考察评估，迈出了关键性一步；四是“双高”糖料蔗基地建设全面铺开，建设面积占全区近40%；五是成功承办第七届广西（崇左）园林园艺博览会，唱响“壮乡情·崇左美”主题；六是工业园区体制改革成效显现，落户项目增多，园区焕发新活力；七是崇左至水口高速公路开工建设，通边达海的综合交通网络加快构建；八是凭祥市通过广西特色旅游名县评估验收，龙州、扶绥、凭祥现代特色农业（核心）示范区获自治区授牌；九是凭祥市和龙州县分别通过国家、自治区义务教育均衡发展评估验收；十是平安建设继续位居全区前列，群众安全感排名全区第一。

随着2015年主要目标任务的完成，我市“十二五”胜利收官，为“十三五”发展奠定了坚实基础。

（一）综合实力跃上新台阶。“十二五”末，全市地区生产总值达到682.82亿元，年均增长9.7%。财政收入75.15亿元，年均增长9.6%。规模以上工业增加值221.78亿元，年均增长14.2%。人均地区生产总值3.34万元，经济发展总体迈进中等收入阶段。

（二）深化改革激发新活力。市本级累计取消、下放和调整行政审批事项365项，目前保留事项242项，53个部门公布了行政审批事项清单，“一窗式”审批让群众办事便捷、效率提速。“先照后证”、“三证合一、一照一码”等商事制度改革落地实施，部分证件当日即可办结。在全区率先开展农村土地承包经营权确权登记颁证试点工作，累计完成土地确权登记12.67万亩，激活了农村土地资源。凭祥口岸关检合作“三个一”改革稳步实施，凭祥海关货物平均通关作业时间由原来约20分钟减少到8分钟左右。创新开展边民互市贸易改革，实现边境贸易货物落地加工增值。园区管理体制改革取得新突破，市城市工业区项目审批效率大大提高，凭祥综合保税区管理体制改革进展顺利，凭祥市与保税区一体化进程加快推进。沿边金融综合改革深入开展，中国（广西凭祥试验区）东盟货币服务平台启动运行，跨境人民币结算量全区第一。全市投资环境总体满意度在全区排名由原来第10位攀升到2015年第3位。改革的深入推进，有效激发了社会创业创新热情和经济发展活力。

（三）开放合作构建新格局。北部湾经济区、珠江—西江经济带、左右江革命老区“三大国家战略”覆盖崇左全境。南宁—崇左—凭祥对外开放经济带上升为国家发展战略，凭祥重点开发开放试验区、中越凭祥—同登跨境经济合作区等纳入国家“一带一路”总体规划，凭祥综合保税区企业进出口总额位列全国35个综保区第12名。对外贸易领先全区，外贸进出口总额连续7年排名全区第一；边境小额贸易进出口总额跃居全国地级市首位，成为中国边境贸易第一大市。区域合作、国际友城深化发展，与国际国内合作日益紧密。今日崇左，已经从边陲“末梢”，发展成为面向东盟开放合作的前沿。

（四）产业转型迈出新步伐。三次产业结构由2010年的29.3:38:32.7优化调整为22.7:40.3:37，第二、第三产业比重分别提高2.3、4.3个百分点。园区建设提档升级，全市有7个园区获国家和自治区认证。产业结构持续优化，糖业产品由原来的白砂糖、酵母、酒精、纸浆4种初级产品增加到精炼糖、酵母抽提物、焦糖色素等11种糖及关联产品。东亚、湘桂、南华等糖业循环经济产业园初具规模，制糖、生物肥、生物发电等多条蔗糖循环经济产业链不断延伸。锰业产品从原来的硅锰合金、电解金属锰2种初级加工产品增加到电解二氧化锰、高纯电解锰粉、锰酸锂等11种中级及以上深加工产品。中铝稀土分离生产线等项目投料试产，南国铜业等项目建设进展顺利，新的工业经济增长点加快形成。红木加工基地一期实现竣工，荣获“国家珍贵树种培育示范市”和“中国红木之都”称号。强优企业不断发展壮大，名牌产品和著名商标大幅增长。产业转型步伐加快，工业化进程加速推进。

（五）项目投资再掀新高潮。覆盖各领域的一批打基础、利长远的项目全方位齐头并进，项目建设规模持续扩大，2015年全市共实施项目2369个，比2010年增加456个，其中超亿元项目75个，比2010年增加18个。项目投资强劲增长，“十二五”期间，全市固定资产投资累计2540亿元，每年增速均高于20%，位居全区前列。崇左至钦州高速公路建成通车，崇左至靖西高速公路加快建设，左江崇左至南宁宋村Ⅲ级航道建成复航，城乡电网不断完善。今日崇左，发展基础更加牢固，发展后劲更加充足。

（六）城乡建设彰显新风貌。城镇化率提高到36.28%，比2010年提高6.48个百分点。中心城区建成区从2010年的22平方公里扩大到32平方公里。城市建设三年大会战、建市十周年献礼工程全面完成，星级酒店、崇左壮族博物馆、崇左人民会堂、“八大住宅区”、园博园等一批城建项目建成使用，城市道路从2010年的46公里增加到121.73公里，中心城市基础设施及绿化、美化明显改善。城市管理体制不断理顺，城市综合管理和服务水平明显提升。南崇城镇带、沿边城镇带建设取得新进展，“书记工程”、特色名镇名村、城乡风貌改造等建设取得良好成效，荣获“广西园林城市”称号。今日崇左，城市建设日新月异，乡村面貌焕然一新。

（七）现代农业呈现新亮点。粮食产量保持稳定，特色种养增产增效。甘蔗种植面积保持在400万亩以上，产糖量保持在200万吨左右，“双高”糖料蔗基地建设效果良好，“中国糖都”地位巩固提升。农业经营方式加快转变，“小块并大块”耕地整治经验在全国推广，累计发展农业企业382家，农民专业合作社1465个，家庭农场56家，均比“十一五”大幅增加。肉牛肉羊规模养殖走在全区前列。甘蔗综合机械化水平全区第一。农业基础设施不断加强，驮英水库及灌区工程项目将于今年实现开工建设。农业经济发展空间更为广阔，农民增收致富渠道不断拓宽。

（八）生态建设取得新成效。节能减排任务全面完成。全部县城建成污水垃圾处理设施，城镇生活垃圾无害化集中处理率88%，污水处理率86%，城市空气质量优良天数比例96.7%。规范河道采砂、网箱养鱼整治效果明显，主要河流监测断面水质达标率、城市集中式饮用水水源地水质达标率均为100%。全市生态环境持续优良，美丽广西乡村建设成效显著，森林覆盖率提高到54.71%，荣获“中国白头叶猴之乡”、“中国木棉之乡”、“广西森林城市”等称号。“发现山水崇左·圆梦别样桂林”旅游发展战略全面打响，被列为首批“全国红色旅游国际合作创建区”，接待游客数量和旅游总收入分别年均增长19%、30%。今日崇左，山更青、水更绿、天更蓝、景更靓、人居环境更美好。

（九）社会事业得到新提升。城镇居民可支配收入和农民人均纯收入分别年均增长9.6%、12.4%，城乡差距进一步缩小。累计投入151亿元实施51件为民办实事工程。落实18.72亿元建设1863个教育项目，全市学前三年毛入园率、义务教育巩固率、高中阶段毛入学率分别提高16个、4个、7个百分点。每万人口发明专利拥有量比2010年增长24倍。18家县级公立医院实施改革，全面实行药品零差率销售，诚信计生基本实现全覆盖。城镇就业、农村劳动力转移就业累计新增10.9万人和29.7万人。城乡居民基本养老保险参保率、新农合参合率、城镇基本医疗保险参保率分别达到98.5%、99.6%、95%。建成农村人饮工程1668处，解决71.13万人口饮水不安全问题。精准识别工作全面完成，累计减贫40.31万人。精神文明、双拥共建、民族团结进步创建深入开展。社会治安综合治理连续6年保持全区第一名，成为广西平安建设的一面旗帜。今日崇左，社会大局和谐稳定，人民群众安居乐业。

（十）政府建设实现新加强。全面推进依法行政，积极建设法治政府。推进政府职能转变和机构改革。106个市直部门公开部门预算和“三公”经费预算。认真执行市人大常委会的决议和决定，自觉接受人大、政协和社会监督，人大代表建议和政协委员提案办结率100%。严格执行“八项规定”，扎实开展党的群众路线教育实践活动和“三严三实”专题教育，深入整治“四风”，促进政府机关和干部作风明显转变，廉政建设和反腐败斗争取得新成效，政府公信力和执行力不断提高。

五年来，我市统计、法制、调处、审计、水库移民、国有资产监督管理、质量技术监督、新闻出版、广播电视、体育、侨务、供销合作、二轻、宗教、涉台事务、机关事务管理、接待、驻邕管理、驻京联络、地震、人防、边防、税务、无线电管理、气象、水文、消防、海关、海事、检验检疫、档案、保密、国家安全、老龄、红十字会、地方志等工作取得新进展。保险、邮政、通信、石油、烟草、盐业等行业和工会、共青团、妇联、工商联、科协、文联、侨联、残联、社科联等群团组织在经济社会发展中发挥了重要作用，作出了积极贡献。

各位代表！“十二五”时期是我市攻坚克难、开拓进取、砥砺奋进的五年，是经济大发展、社会大进步、民生大改善的五年。“十二五”的发展证明，做好政府工作必须坚持改革开放，以改革促进开放，以开放倒逼改革，不断增强发展动力活力；必须坚持积极作为，务实推进“四大建设”、“五个打造”、“两加”“两成”，积极寻求新突破，推动新发展；必须坚持绿色发展，既要金山银山，更要绿水青山，走绿色生态、可持续发展之路；必须坚持稳中求进，主动适应经济发展新常态，确保调速不减势、量增质更优；必须坚持依法行政，加快职能转变，建设人民满意的法治政府；必须坚持民生至上，让发展成果更多地惠及广大群众，赢得人民信赖与支持。

各位代表！五年成就来之不易。这是自治区党委、政府和市委正确领导的结果，是历届党委、人大、政府、政协打下良好基础的结果，是全市人民群策群力、共克时艰的结果，是社会各界大力支持帮助的结果。在此，我谨代表市人民政府，向全市各族人民，向人大代表、政协委员，向各民主党派、工商联、无党派人士、各人民团体，向驻崇左人民解放军、武警部队官兵，向所有关心支持崇左发展的社会各界，表示崇高的敬意和衷心的感谢！

同时，我们也清醒地看到，受多重因素影响，我市发展中仍有不少矛盾和困难，主要是：经济总量不大，经济结构不合理，工业基础仍然薄弱，与全国全区同步全面建成小康社会压力较大；土地、资金等要素供需矛盾突出；公共资源配置不尽合理，在入学、就医、养老等方面与群众的需求仍有差距；全市仍有34.18万贫困人口，补齐民生短板和脱贫攻坚任务艰巨；政府职能仍需加快转变，各领域改革工作有待进一步深化。对于这些矛盾和困难，我们将积极采取有效措施，努力加以克服和解决，决不辜负全市各族人民的期望。

二、 “十三五”的奋斗目标和主要任务

“十三五”时期是我市与全国全区同步全面建成小康社会的决胜期，是加快构建“一带一路”重要门户前沿城市、实现“两加”“两成”的关键阶段。我们要紧紧把握“一带一路”建设重大机遇，充分发挥“三区叠加”政策优势，抢机遇、再开放、强落实，再谱崇左发展新篇章。

根据《中共崇左市委员会关于制定国民经济和社会发展第十三个五年规划的建议》，市人民政府在广泛征求人大代表和政协委员、各地各部门以及社会各界意见的基础上，制定了《崇左市国民经济和社会发展第十三个五年规划纲要（草案）》（以下简称“《纲要草案》”），整个起草过程充分体现了科学、民主决策的要求，是集体智慧的结晶。《纲要草案》共分3大板块15篇58章。导语和第一篇构成第一板块，分析“十三五”发展环境，明确指导思想、基本原则和主要目标。第二篇至第十四篇构成第二板块，就经济社会发展各领域进行全面部署。第十五篇为第三板块，提出保障措施。在这里向各位代表着重报告三个方面内容：

（一）关于“十三五”发展总体要求。

加力开放合作，加快南崇经济带建设，把崇左建设成为与全国全区同步全面实现小康社会、成为面向东盟开放合作的新高地，这是市委“十三五”规划建议提出的“两加”“两成”目标。根据这个目标，我市“十三五”发展的总体要求是：全面贯彻党的十八大和十八届三中、四中、五中全会及自治区党委十届六次全会和市委三届六次全会精神，按照“四个全面”战略布局的要求，根据自治区党委“两个建成”的目标要求，抢抓新机遇，奋力大发展，着力发展五大支柱产业，打好十大重点战役，实现“两加”“两成”任务目标。

这一总体要求，主要是围绕中央“四个全面”战略布局、“五大发展理念”和自治区“三大定位”、“两个建成”，结合新形势和我市发展实际，强化对国家、自治区战略的落实，确保中央、自治区党委决策部署在崇左落地生根。

（二）关于“十三五”发展目标。

根据上述总体要求，《纲要草案》提出了经济发展、创新驱动、民生福祉、资源环境4大类30项发展目标，其中最主要的综合性指标是地区生产总值年均增长8%以上，城乡居民人均收入年均增长9%。提出这一目标，主要基于以下考虑：一是既要确保翻番的要求，更要缩小发展差距。根据测算，到2020年我市地区生产总值和居民收入实现比2010年翻一番，“十三五”需分别年均增长4.4%、7.2%。从发展基础条件看，实现这个速度没有问题。但我市作为后发展欠发达地区，需要进一步缩小与全国全区平均水平的差距，跟上全国全区发展步伐，必须把握“努力实现一个高于全区平均水平的增长速度”的原则，保持必要的增长速度。为此提出地区生产总值年均增长8%以上，争取高于全国1.5个百分点、全区0.5个百分点。二是既要考虑发展潜力，也要考虑支撑条件。要实现8%以上的年均增长目标，从支撑条件看，一、二、三产需分别年均增长3%、8.5%、11%。根据测算，“十三五”期间我市经济潜在增长率在7.5-8.5%之间，一、二、三产有望分别增长3.3%、10%、12%。从发展趋势看，固定资产投资年均增长17%，外贸进出口总额年均增长13%，社会消费品零售总额年均增长10%，都是有条件、有基础的，经过努力是可以实现的。三是既要稳增长，又要加快调结构。未来五年，新常态经济发展面临速度换挡、结构调整、动力转换等节点，“五大发展理念”对发展提出了更高要求，必须改变过去粗放型的发展方式、增长方式，注重从创新驱动着力，加快转方式、调结构，提高经济发展质量和效益。要统筹稳增长与调结构的关系，追求经济增长要为调结构、促转型、推进结构性改革留出空间。

综合考虑“十三五”时期我市发展环境、政策导向、增长潜力、发展趋势，提出地区生产总值年均增长8%以上的目标，统筹兼顾了发展速度、发展质量、发展方式等方面要求，总体上是科学合理、积极可行的，有利于把着力点引导到结构性调整、提质增效上来，实现有质量、有效益、可持续的增长。

（三）关于“十三五”重点任务。

根据市委关于“十三五”规划建议，《纲要草案》提出坚持“五大发展理念”，狠抓创新驱动战略、开放带动战略、双核驱动战略、绿色发展战略、三大攻坚战等“五个落实”，发展“糖矿贸红绿”五大支柱产业，打好基础设施战役、产业园区战役、脱贫攻坚战役、老区振兴战役、甘蔗“双高”战役、矿业开发战役、红木产业战役、文化旅游战役、边境贸易战役和新型城镇化战役等“十大重点战役”，着重抓好以下重点任务。

——建设面向东盟开放合作新高地。构建服务“一带一路”战略的全方位开放格局，形成陆路东盟大通道、西南地区出海大通道，建立以崇左为主中心，以扶绥、凭祥为副中心，以南宁—崇左—凭祥对外开放经济带、沿边经济合作和边境新型村镇建设带为轴线，辐射崇左市域全境的“三核两带”开放发展空间骨架。建立健全开放合作平台，大力建设凭祥重点开发开放试验区，加快推进中越凭祥-同登跨境经济合作区、凭祥综合保税区、中泰（崇左）产业园等开放合作平台，提升开放服务水平。把崇左培育成为政策活、通道畅、集聚力强、辐射面广的增长区域。

——大力推进产业优化升级。围绕“糖矿贸红绿”五大支柱产业，不断优化产业结构。糖业方面，促进糖业“二次创业”，科学合理布局、高标准建设200万亩“双高”糖料蔗基地。推动制糖业技术改造升级，力争建成全国重要的糖产品精深加工基地。加快建设崇左食糖交易中心。矿业方面，推进锰业高端化、生态化发展，建设南国铜循环产业园、广西重要新型稀土材料产业基地，加快生态铝产业开发利用。边境贸易方面，深入实施加工贸易倍增计划，构建崇左百里边境加工贸易带，提升和扩大对外贸易。红木产业方面，推进珍贵树种培育示范带建设和红木产业加工基地建设，积极发展林木加工产业。绿色产业方面，大力实施“发现山水崇左·圆梦别样桂林”旅游发展战略，积极融入广西旅游“三区一带”发展格局，建设边关风情旅游带核心区。发展新能源产业、新型装备制造业、生物制药产业等新兴产业。逐渐摆脱经济支柱单一、产业层次低的困境，加快开辟多业并举、创新发展的新路子。

——提速发展现代服务业。大力发展商贸服务业，完善城乡商贸服务体系。培育以电子商务为主的“互联网+”经济，建设跨境电商产业园。大力拓展“互联网+”应用领域，推动“互联网+”医疗服务、教育培训等领域拓展。做强金融保险业，务实推进沿边金融服务领域开放，创新开展跨境人民币业务。积极发展健康养老产业等服务业，加快建设4个自治区服务业聚集区，培育20个以上市级服务业聚集区。加快形成新经济形态，拓展发展新动能，打造经济增长新一极。

——加快农业现代化步伐。稳定粮食播种面积在180万亩、总产量53万吨左右。打造“一乡一业”、“一村一品”特色产业，加快建设现代特色农业（核心）示范区。扩大与东盟农业合作。推进农业产业化经营，加大“小块并大块”耕地整治力度，推动土地使用权流转，形成家庭农场、专业合作社、种养大户和龙头企业多元经营主体发展新格局。完善支农惠农政策，不断提升农业机械化水平，积极发展政策性农业保险和“互联网+”农业。建设特色明显、示范引领的优势区域。

——打好新型城镇化战役。以人的城镇化为核心，合理定位中心城市、大县城和特色城镇带的功能，提升城镇化发展质量。“十三五”期间，实现城镇新增人口28万人，全市常住人口城镇化率达到46%。强化中心城市集聚辐射功能，打造面向东盟的国际区域性中心城市，到2020年，中心城市人口达到30万人。实施大县城战略，打造凭祥、扶绥两个副中心城市，支持大新、宁明、天等、龙州四县推进县域城镇化。统筹城乡一体化发展，构建个性鲜明、功能完善、城乡互动、生态宜居的城镇化发展新格局。

——打好基础设施建设战役。完善综合运输网络，力争到2020年全市铁路总里程达到370公里，公路总里程突破8000公里，实现1000吨级航道直通粤港澳，建成2个以上通用机场，构建东西轴线、南北轴线、左江国际航运带、通用机场多节点的综合交通运输骨架。完善民生水利基础体系，提高城乡水利防灾减灾能力。加强水资源工程建设，确保城乡供水安全。加快信息基础设施建设，进一步提高全市网络覆盖率和传输能力。构筑基础更牢、效率更高的新优势。

——打好脱贫攻坚战役。推进特色产业富民、扶贫移民搬迁、农村电商扶贫、教育劳务创业、基础设施建设、科技文化医疗扶贫、金融扶贫、社会扶贫、生态补偿和产权收益脱贫、政策兜底和关爱服务等脱贫攻坚“十大行动”，提前一年完成脱贫“摘帽”，到2019年34.18万贫困人口全部脱贫，返贫率控制在3%以下，287个贫困村、4个贫困县全部脱贫摘帽。健全“4321”帮扶机制，实现帮扶对象全覆盖。全面完成脱贫攻坚任务，与全国全区同步进入小康社会。

——保障和改善民生福祉。大力发展教育事业，促进教育协调发展。实施“全民健康”重大战略，打造“健康崇左”。坚持计划生育基本国策，实施“全面两孩”政策。促进体育事业发展。深入实施就业优先发展战略，城镇登记失业率控制在4.2%以内。拓宽城乡居民增收渠道，提高城乡居民收入水平。完善社会保障、社会救助和福利体系，推进机关事业单位养老保险制度改革，实施全民参保登记计划。健全城乡住房保障体系，“十三五”期间建设城镇保障性住房2.3万套。把崇左建设成为人民安居乐业、百姓安康幸福的国门福地。

——彰显文化繁荣发展实力。培育和践行社会主义核心价值观，加快形成崇左城市精神。深入开展文明城市（县城）、文明村镇、文明单位创建活动。加强社会征信制度建设，着力打造“诚信崇左”。提高公共文化供给能力，实施文化惠民活动。加强文化遗产保护管理，推进花山申遗工作，加快太平府故城修复保护。培育文化产业主体，加大文化创意。深度开发骆越根祖文化等内涵和价值，促进文化产业化发展。以先进文化美化全市人民干事创业的精神家园，以高尚精神鼓舞全市人民攻坚克难成就大业。

——加快建设生态宜居家园。实施主体功能区战略，强化空间布局引领。大力发展生态经济，用循环经济理念指导产业结构调整，推进产业生态化发展。建设资源节约型社会，加强资源节约与高效利用，大力推进节能减排。加强生态建设和环境保护，加大对重点领域的生态保护，实行最严格的环保执法制度。加大环境治理和监测，从源头上防治污染和保护生态。不断探索全市上下热爱美好家园、呵护美好家园、建设美好家园的新路子。

——释放全面深化改革红利。推进供给侧、需求侧双向发力，扩大要素供给，提高经济增长质量和效益。优化需求侧管理，保持投资稳定增长。深化投融资体制改革，充分发挥市场在资源配置中的决定性作用。创新政府和社会资本合作机制，激活社会资本投资活力。深化行政体制改革，加快建立健全权力清单、责任清单。深化商事制度改革，推进“证照分离”改革等工作。深化行政机构改革，探索建立行政审批局。深化财税金融体制改革，建立全面规范、公开透明预算制度。加快推动沿边金融综合改革，推动开展跨境人民币业务。深化国有企业改革，支持民营经济发展。以改革释放更多红利，激发社会活力，为科学发展提供体制机制保障。

——提升创新驱动发展推力。加强科技体制机制建设，强化企业创新主体地位，构建以企业为主体、市场为导向、产学研相结合的技术创新体系。提升创新能力水平，制定完善加快实施创新驱动发展的政策，建设一批知识产权优势企业。深入实施人才强市战略，统筹推进各类人才队伍建设，积极引进高层次人才，优化人才队伍结构；完善招才引智平台，健全人才工作机制。让人才充分施展，让智慧充分涌流，形成千帆竞发、百舸争流的创新发展新局面。

——促进边疆和谐稳定发展。推进法治政府、法治社会建设。加强民主政治建设，自觉接受监督。加强廉政建设，坚决纠正损害群众利益的不正之风。不断深化平安崇左建设，积极推进社会治理创新，保障人民群众生命财产安全。推进边疆和谐建设，深入实施“爱民固边”工程，健全国防动员体系。深入开展民族团结进步创建活动。努力营造富民兴边、富民强边的发展环境。

各位代表！“十三五”发展蓝图已经绘就，前进号角已经吹响。全市各族人民团结一致、万众一心，击鼓奋进、顽强拼搏，一定能够与全国全区共同迈入小康社会，开创更加美好的明天！

三、 2016年的主要目标与重点工作

今年是“十三五”的开局之年，也是全面建成小康社会决胜阶段的开局之年。今年我市经济社会发展的主要预期目标是：地区生产总值增长8.5%，财政收入增长7%，规模以上工业增加值增长10%，固定资产投资增长20%，社会消费品零售总额增长10%，进出口总额增长13%，居民人均可支配收入增长9%，居民消费价格涨幅控制在3%以内，城镇登记失业率控制在4.2%以内，城镇新增就业1.4万人。以上目标，主要是从“十三五”目标和全面实现小康社会的大背景以及今年经济形势更为严峻复杂的情况出发，从积极稳妥、统筹兼顾等方面考虑，确保年度目标在合理区间内与阶段目标协调一致。在实际工作中，我们将全面贯彻落实“五大发展理念”，狠抓“五个落实”，打好“十大重点战役”，提振发展信心、积极主动作为，努力实现“十三五”开好局、起好步。我们将重点抓好以下工作：

（一）狠抓全面深化改革，增强发展动力活力。

积极推动结构性改革。按照优化存量、引导增量、有效减量的要求，加快去产能、去库存、去杠杆、降成本、补短板，推进供给侧结构性改革，提高全要素生产率。去产能方面，加快企业兼并重组，加大力度淘汰落后产能，严格控制增量，防止新的产能过剩。去库存方面，推进户籍制度改革，加快农民工市民化，建立购租并举的住房制度，实施公廉并轨管理制度，推进棚户区改造货币化安置和保障性安居工程“以租代建”、“以购代建”模式相结合，实施11841套棚户区改造，促进商品房去库存量。降成本方面，落实减税降费让利等政策，降低企业生产经营成本。

加快政府职能转变。进一步加大简政放权力度，最大限度减少审批环节和审批事项，4月底前公布政府部门权力清单和责任清单。深化商事制度改革，推进电子营业执照和全程电子化登记管理。加强各级政务服务中心建设，开工建设市电子政务公共服务体系平台项目。继续落实公务用车制度改革，筹备开展事业单位、国有企业公务用车制度改革工作。

深化投融资体制改革。推进投资项目在线并联审批监管平台建设，争取年内上线运行。支持发行企业债券，为项目建设提供稳健资金支持。加强政府性债务管理，规范发展政府投融资平台。加强市公共资源交易平台管理，探索建立县级公共资源交易中心。

深化财税金融体制改革。深化预算管理制度改革，进一步加大预决算公开力度，完善政府预算体系，深入推进“营改增”改革，探索中期财政规划管理。加快沿边金融综合改革，继续深化跨境人民币结算业务，在凭祥市建设互市贸易结算中心。

深化相关体制机制改革。深化农村改革，在6个县（区）、33个乡镇、327个村（社区）开展农村土地确权登记颁证工作，加快土地流转和“小块并大块”耕地整治。加快园区管理体制改革，推进中国—东盟青年产业园扩区及管理体制改革，推动市城市工业区体制改革落实到位，并扩大到市、县两级各园区。深化华侨农林场体制改革。统筹推进开放合作、国有企业、供销合作社等其他领域改革。

（二）狠抓开放带动，打造对外开放升级版。

搭建高水平开放合作平台。加快南宁—崇左—凭祥对外开放经济带建设，推进凭祥重点开发开放试验区、龙州边境经济合作区申报和建设，推动中越凭祥—同登跨境经济合作区尽快获得两国政府批准建设。积极配合北部湾自由贸易试验区崇左区块申报建设。加快凭祥综合保税区二期建设，推进凭祥市与保税区一体化发展。加快推进中泰（崇左）产业园和中越德天·板约跨国旅游合作区建设，促进跨境产业合作。

大力发展口岸经济。加强口岸、边民互市点基础设施建设，推动爱店一类口岸通过国家验收。继续推进硕龙、科甲、平而关口岸升格和友谊关、水口口岸扩大开放，配合推进北部湾“六市一关”工作和广西国际贸易“单一窗口”建设，提高通关效率。打好边境贸易战役，培育对外贸易优势企业。实施凭祥浦寨（弄怀）、龙州水口边民互市区试点改革，进一步扶持发展壮大边民互助组，通过“互助组+边民+企业+金融”等运作模式开展互市贸易。探索发展旅游贸易和市场采购贸易，扩大对外贸易份额。深入实施加工贸易倍增计划，推动通道经济向口岸经济转型升级。

拓展区域合作空间。主动融入“一带一路”建设，大力实施“双核驱动、三区统筹”战略，在服务国家、自治区战略中提升开放型经济发展水平。加强与东盟等国家交流合作，加快国际产能合作，主动承接商贸、金融、高新技术等产业转移。继续开展国际友城合作交流，拓展对外开放合作渠道。实施精准招商，力争全市区外招商引资项目到位资金260亿元以上。

（三）狠抓转型升级，加速新旧动能转换。

大力推进创新驱动发展。积极参与实施中国制造2025、"互联网+"行动。突出企业创新主体地位，引导支持企业进行技术、产品、业态等方面创新，加快培育发展一批名牌产品及高新技术示范企业。启动和推进自治区级崇左锰及新材料高新区建设，完善广西锰业千亿元产业研发中心等自治区级创新平台，培育扶持中粮糖业、中铝稀土等市级创新平台。

推动园区提质发展。打好产业园区战役，加快完善园区道路、供水供气、排污等基础设施和标准厂房建设，不断提升园区承载能力。加大南崇经济带沿线园区建设力度，明确各园区产业定位，引导项目向园区集中发展，培育优势产业集群。

稳定和提升传统优势产业。糖业方面，大力实施糖业"二次创业"，发展蔗糖循环经济和深加工，重点推进金冠等项目竣工投产，力争崇左东亚生物质能发电、湘桂活性干酵母工程等项目开工建设。锰业方面，打好矿业开发战役，改造提升生态型锰精深加工，推动中信大锰镍钴锰酸锂三元材料生产线等项目竣工投产，推进新振锰业中低碳锰铁合金生产等项目加快建设，推进中信大锰天等电解金属锰扩建技改项目、布东产业园动力车间项目上半年开工建设。红木方面，打好红木产业战役，深入推进珍贵树种培育示范带建设，推动红木产业基地一、二期36个项目全部投产；加快发展红木电商，扩大红木销售渠道。建材业方面，推动东泥水泥项目竣工投产，力争南方水泥专用码头项目10月前开工建设。

培育发展新产业。加快南国铜业项目建设，力争年内竣工投产。加快龙州氧化铝、龙州中恒万华不锈钢等项目前期工作，力争年内开工建设。推动中铝稀土分离生产线项目扩大生产，力争中铝稀土催化剂项目开工建设。大力发展林产业，推进龙赞东盟国际林业循环经济产业园一期8个项目竣工投产，二期18个项目开工建设。加快发展坚果加工产业，推动昆仑物流坚果加工等项目加快建设。

培育和引进强优企业。引导和支持现有企业通过技术改造、兼并重组、招商引资等方式，加大对中小企业的扶持，培育发展和引进一批优势企业。力争到今年底，全市规模以上工业企业达到160家以上，拥有主营业务收入超亿元企业90家以上，超10亿元企业14家以上，超30亿元企业2家以上。

提速发展现代服务业。加快建设服务业发展平台，推动凭祥综合保税区等4个服务业集聚区建设取得突破。加快电子商务服务体系建设，推动跨境电子商务健康快速发展。大力发展现代物流业，加快中越边境中药材商贸物流中心等项目建设。推动崇左市农副产品交易中心上半年竣工使用，加快中国-东盟（凭祥）农副产品专业市场等项目建设，打造一批城市综合体、购物中心和特色商业街区。大力发展生态服务业，加快推动中国乐养城建设。优化消费市场环境，力争年内实现新增限上企业30家，全年社会消费品零售总额超130亿元。

加快发展现代农业。狠抓粮食生产，确保粮食播种面积稳定在183万亩、粮食总产量50万吨以上。坚持甘蔗生产不动摇，开展糖料蔗价格指数保险试点工作，提高种蔗积极性，力争甘蔗种植面积达420万亩。打好甘蔗"双高"战役，确保完成年度36万亩"双高"糖料蔗基地建设任务。加快现代特色农业（核心）示范区建设，推动区、市、县、乡四级示范区规划创建。大力发展"菜篮子"、水果、中药材、养殖等特色效益农业。积极发展休闲生态观光农业，培育光伏农业、农产品定制等新业态。大力培育新型农业经营主体，力争年内新增农民专业合作社100家以上、家庭农场16家以上。

（四）狠抓投资拉动，夯实经济社会发展基础。

打好项目前期工作攻坚战。市本级财政安排5000万元以上资金作为重大项目前期工作经费，加快推动一批项目前期工作，尽快实现开工建设。打好老区振兴战役，积极谋划争取更多的重大项目、重大工程、重大事项纳入国家和自治区规划盘子。

全力推动重大项目建设。组织推进市层面统筹推进134个重大项目的建设工作，努力扩大有效投资，力争完成投资150亿元。统筹用好各类资金，支持重点产业、重大基础设施和重点区域、重点领域发展。打好基础设施战役，着力推进一批重大基础设施项目建设。公路方面，推动崇左至靖西高速公路、德保至天等二级公路年内建成通车；推进崇左至水口高速公路、南宁新江至崇左扶绥一级公路、驮卢至大新、大塘至渠黎二级公路等项目加快建设；推动水口至驮隆中越界河公路二桥等跨境交通项目尽快开工建设；加快隆安至硕龙高速公路前期工作，推进崇左至爱店高速公路规划论证工作。大力推进农村公路建设，力争年内实现100%建制村修通水泥（沥青）路。铁路方面，力争湘桂铁路南宁至凭祥段扩能改造项目今年开工建设。水运水利方面，推动扶绥将军岭作业区一期工程实现正常运营，加快崇左港中心港区濑湍作业区建设。加快渠珠水利枢纽工程、左江治旱黑水河灌区等项目前期工作，力争驮英水库及灌区工程项目6月前开工建设。机场建设方面，加快推进大新、龙州通用机场前期工作，力争早日开工建设。能源方面，推动崇左市110KV电网、天等县牛头岭风电场等项目开工建设，加快推进农网升级改造工程建设。

（五）狠抓城镇化建设，促进城乡统筹发展。

科学编制城乡规划。推进崇左市城市总体规划修编和中心城区抗震防灾、保护性建筑等专项规划编制，完善各县（市）总体规划修编和控制性详细规划编制。加快特色乡镇规划编制，优化城镇空间布局和规模结构，加强重点区域城市设计，提高规划建设水平。

加快中心城市建设。打好新型城镇化战役，继续做好园博园完善、运营等工作，提升园区建设管理水平。加快崇左大桥、市体育中心、环城西路、天然气管道工程等项目建设，推动龙腾湖风情街一期、金融中心竣工使用。加大旧城区改造力度，推动左江两岸综合整治工程上半年开工建设。大力发展公共交通，提升城市服务功能。推进国家级园林城市创建工作。力争中心城区建成区面积增加2平方公里以上，全市城镇化率提高1.5个百分点。

抓好县城和特色城镇建设。加快县城扩容提质，不断增强县城聚集能力。积极融入国家口岸城镇建设体系，促进中心城市与口岸城镇联动发展。抓好凭祥市全国中小城市综合改革试点城市建设工作。加快桐棉镇、堪圩乡、水口镇、夏石镇4个边境乡镇特色新型城镇化建设，深入实施山圩镇、渠旧镇、下雷镇、雷平镇、水口镇、驮卢镇6个广西百镇建设项目。继续抓好乡土特色建设示范等工作。

完善城市管理服务。深入推进文明城市创建工作，不断提高城市文明水平。积极推动城市执法体制改革，提高城市管理和公共服务水平。深入开展"两违"综合治理，不断改善市容市貌。

（六）狠抓生态建设，推动绿色发展。提升旅游品牌。大力实施"发现山水崇左·圆梦别样桂林"旅游发展战略，打好文化旅游战役。继续推进左江花山岩画文化景观申遗工作，确保申遗成功，打造世界级文化旅游品牌。推进边关风情旅游带建设，大力发展红色旅游，加快重大旅游项目建设，确保扶绥龙谷湾景区年内开园运营。加大旅游资源整合开发力度，进一步融入北部湾大旅游圈，继续推进中越友谊关-友谊国际旅游合作区建设。继续抓好特色旅游名县、名镇创建和A级景区创建工作，确保大德天景区成功创建5A级景区。力争年内接待游客总人数和旅游总收入分别增长11.8%、20%。

加强生态建设。加强白头叶猴自然保护区等生态涵养区和林地资源保护，增强生态承载能力。加强造林绿化，力争森林覆盖率提高到54.75%，成功创建"国家森林城市"。深入推进美丽广西乡村建设，建成自治区级示范村屯绿化265个，一般村屯3075个。让青山常在，让绿水长流，为崇左发展留下宝贵绿色财富，更好造福子孙后代。

强化环境治理。加强环保基层能力建设。全面加强大气污染防治和环境监测，开工建设一批乡镇、村屯污水处理、垃圾处理设施项目等。强化节能减排工作，进一步控制污染物排放。深入开展环保专项整治，杜绝重特大环境污染事件发生。继续巩固河道采砂、网箱养鱼管理工作，建立健全长效机制，推动常态化管理。

（七）狠抓精准扶贫，打好脱贫攻坚战役。

强化精准管理。充分运用精准识别成果，加快建立扶贫大数据管理平台，市县乡村四级建立"挂图作战、清单管理、滚动集成、精准摘帽"精准管理模式，实现扶贫对象信息科学化、精准化和动态化管理。

抓好精准施策。全力推进脱贫攻坚"十大行动"，确保全年减贫9万人、29个贫困村脱贫"摘帽"。实施产业扶贫，在贫困县培育5个以上、面上县培育2个以上覆盖面广、辐射带动能力强的优势特色产业。加快推进边贸扶贫，增加边民经营性收入。大力发展电商扶贫，通过电商推销农产品增加收入。实施"雨露计划"扶贫培训，提升扶贫对象自我发展能力。加快扶贫生态移民搬迁工作，年内完成搬迁2938人。加快扶贫综合示范点建设，各县（市、区）建设1-2个示范点。

创新精准帮扶。每个县（市、区）开展2个或2个以上资产性收益扶贫试点，鼓励将财政专项扶贫资金和其它涉农资金投入设施农业、养殖、乡村旅游等项目，增加群众收入。深入推进"百企帮百村"活动，实现287个贫困村"村企共建"全覆盖。强化扶贫责任，落实"4321"干部帮扶机制，动员全社会参与精准帮扶。

（八）狠抓公共服务，持续增进人民福祉。

优先发展教育事业。深入实施教育"双百"工程，筹措3亿元以上资金推进"以教促增"项目建设。积极实施第二期学前教育三年行动计划。大力推进义务教育均衡发展，推动龙州县通过国家验收，宁明、大新、天等通过自治区级评估验收。全面实施基础教育质量提升工程，推进义务教育学校标准化建设和普通高中建设，推进民师院附属中学创建自治区示范性高中。继续深化职业教育教学改革。

促进大众创业就业。落实创业就业政策，扎实开展创业培训，提升大众创业能力。统筹做好高校毕业生、城镇就业困难人员等各类群体就业工作，新增农村劳动力转移就业2.8万人。推进跨境劳务合作，打造边境劳务合作示范区。

完善社会保障体系。推进社会保险基金征缴和"全民参保登记"工作，城乡居民基本养老保险参保率达98%以上。加快全市社会保险"五险合一"和城乡低保信息系统建设。加快推进机关事业单位养老保险制度改革工作。进一步完善城乡最低生活保障制度，统筹扶贫标准和低保标准有效衔接，推动社会保障由制度全覆盖到人群全覆盖。进一步加强空巢老人和留守儿童关爱服务保障工作。实施300个农村饮水巩固提升工程，改善9万人农村居民饮水条件。

加快发展卫生计生文体事业。深化医药卫生制度改革，全面推进县级公立医院综合改革。坚持计划生育基本国策，稳妥有序实施"全面两孩"政策，打造诚信计生升级版。继续推行文化惠民政策，抓好村级公共服务中心和广播电视村村通工程建设，完成100个农村电影院建设。深入开展群众体育活动。

加强和创新社会治理。加快社会治安立体化防控体系建设，实行网格化管理、社会化服务，健全基层综合服务管理平台。加快社会稳定风险评估机制建设。严密防范和依法惩治各类违法犯罪活动，严厉打击涉恐涉枪涉毒走私等跨国、跨区域犯罪活动。健全矛盾纠纷精准排查和精细化解机制，加强信访、安全生产、食品药品安全监管等工作。加强社会信用体系建设，推进崇左信用信息平台建设。

继续实施为民办实事工程。筹措资金50亿元，抓好社保、健康、教育、水利、扶贫、安居、农补、生态、文化、交通等一批惠民项目。

各位代表！民族团结是各族人民的生命线，大力推进民族团结进步创建活动，巩固民族团结。加强宗教工作，依法管理宗教事务，促进宗教和谐。加强人才工作，抓好人才小高地提升工程，积极引进一批急需紧缺专业高层次人才。推动军民深度融合发展，做好国防教育、民兵预备役等工作，加强国防动员、国防后备力量建设和人民防空工作。深入开展双拥共建，加快全国双拥模范城创建工作。发展老龄事业，依法保障妇女儿童和残疾人的合法权益，支持工会、共青团、妇联等群团组织开展工作。

各位代表！做好今年和"十三五"时期经济社会发展各项工作，必须以新的面貌、新的作风加强政府自身建设，尽快提高新常态下推动发展的能力和水平。坚持依法行政，强化依法执法，不断提高行政效能和服务水平。自觉接受人大及其常委会监督和人民政协民主监督，定期向人大及其常委会和政协报告工作，切实做好人大代表建议和政协委员提案办理工作。认真听取各民主党派、工商联、无党派人士、人民团体和社会各界人士的意见，健全依法决策机制，强化决策监督，推进责任政府建设。全面推进政务公开，提高政府公信力和执行力。全面落实从严治党要求，巩固"三严三实"专题教育成果，进一步改进作风，形成长效机制。严格落实党风廉政建设主体责任，履行好"一岗双责"，持之以恒纠正"四风"，坚持不懈推进党风建设和反腐败工作。

各位代表！新形势催人奋进，新目标鼓舞人心。让我们紧密团结在以习近平同志为总书记的党中央周围，在市委的领导下，改革创新、锐意进取，团结奋进、真抓实干，向着与全国全区同步全面建成小康社会的目标奋勇前进！
